# Bioaccumulation of Organophosphorus (OPs) and Carbamate (CBs) Residues in Cultured Pangas Catfish (*Pangasius pangasius*) and Health Risk Assessment

**DOI:** 10.1155/2022/4644227

**Published:** 2022-10-26

**Authors:** G. M. M. Anwarul Hasan, Anuj Kumer Das, Mohammed A. Satter, Md. Asif

**Affiliations:** ^1^Institute of Food Science and Technology (IFST), Bangladesh Council of Scientific and Industrial Research (BCSIR), Dr. Qudrat-E-Khuda Road, Dhaka 1205, Bangladesh; ^2^Hi-Tech Health Care Ltd., Banani, Dhaka-1213, Bangladesh

## Abstract

In the present study, the presence of organophosphorus (OPs) and carbamates (CBs) residues in the pond water and cultured Pangas catfish (*Pangasius pangasius*) samples collected from Comilla and Mymensingh areas were detected and assessed for their potential health risks. A total of 100 samples from each category were analysed among which 17% of the pond water samples and 9% of the fish samples were detected contaminated with OP and CB residues. The pond water and fish samples were extracted by liquid-liquid extraction (LLE), quick, easy, cheap, effective, rugged, and safe (QuEChERS) extraction, and ultrasonic extraction, respectively, and analysed through gas chromatography tandem mass spectrometry (GC-MS/MS). Among the detected OPs, Dursban (chlorpyrifos) and dichlorvos were detected, while among CB pesticides, carbofuran and sevin (Carbaryl) were detected in fish muscle samples. The detected OP and CB residual levels were below than the maximum residue limits (MRLs). The risk assessment study indicated no potential health risks. However, the level of compliance should be maintained through proper monitoring and controlling the overuse of pesticides in agricultural fields for public health safety.

## 1. Introduction

Pesticides are rapidly becoming the most valuable stuffs in cultivation. Though their contribution is in the enlargement of harvest yield, many other living organisms are adversely affected by their improper uses. Environmental disruption is becoming more common as a result of ecosystem instability. The health of Aquatic species llike fish deteriorates by disturbing their metabolic functions remarkably. Because of prolonged exposure, pesticides can cause tumour growth, the development of a malformed organism, genetic modification, blood disorders, endocrinological dysfunction, and genotoxicity [1].

Exposure of pesticides to humans has been responsible for the development of different types of diseases such as cardiovascular diseases, dementia, hypertension, cancer, and so on. [2]. Dysfunction of cholinesterase activity in the nervous system via blocking nerve transmission at synapses in cholinergic neurons is one of the potential interruptions for both carbamate (CB) and organophosphate (OP) pesticides [3]. Specifically, organophosphates block the action of cholinesterase (ChE) enzymes, red blood cell (RBC), ChE, and serum ChE, allowing organophosphate poisoning to have cholinergic indications[4]. Pesticides are also known as EDCs (endocrine disrupting chemicals) which are chemicals that interfere with the normal functionality of the endocrine system including nontarget species [5]. The use of ED pesticides has been linked to the increased prevalence rate of disease and malformations in humans and wildlife. This pesticide class has been linked to lowering insulin secretion, particularly in exposure to glucose. By reducing mitochondrial ATP synthesis, both OP and CB lead the way for hyperglycemia, which results in the breakdown of glycogen, lipids, and finally proteins to provide energy. They can also modify the enzymatic pathways involved in the generation of glucose from proteins, lipids, and glycogen in cells including the liver and muscle. Because of poor insulin secretion, this hyperglycemia cannot be diminished [6]. In rats and mice, the organophosphate pesticide chlorpyrifos (CPF) has been shown to cause neurological damage, malformation and micronucleus development, persistent developmental disabilities, and maternal toxicity. As a consequence, CPF is a highly genotoxic chemical that causes DNA damage and cell apoptosis [7]. Abnormalities of the central and peripheral nervous systems, as well as cardiovascular diseases, have been observed following a single massive dose of organophosphates (OPs) [8]. The potential influence of long term exposure to OP and CB pesticides on male reproductive function may result in sperm chromatin damage, diminished semen quality, and hormonal changes, leading to poor reproductive health outcomes [9]. A population-based study found that putative connections between pesticide exposure and human health raised the incidence of nonlymphoma Hodgkin's and dementia [10–12]. Bangladesh is known as the country of rivers. In the agricultural sector, rivers play a vital role in cultivation. The use of excessive pesticides in the farmlands near waterways has a substantial negative impact on streams, which are becoming highly polluted. Water quality has drawn significant attention as one of the most critical environmental issues in developing countries; particularly, Bangladesh is confronting a major water pollution crisis. By evaluating health risk assessment of pesticides in the water bodies, the water quality in many areas of the country has already degraded, and a substantial number of people are at risk of water pollution [13]. According to the recent study, high quantities of diazinon, carbofuran, and carbaryl pesticide residues in water bodies pose a serious threat to the aquatic ecosystem and public health [14]. Another study indicated the contamination of several river water bodies and fishes through pesticides in Bangladesh [15]. Not only the river water is contaminated with pesticide residues but also vegetables, fruits, animal feed, milk, fish, and egg were contaminated with pesticides [16, 17] Therefore, pond water and cultured fishes should also be monitored for detection of pesticide residues.

Fish is considered as a balanced diet worldwide because of its nutritional values [18]. Fish contributes about 60% of animal protein demand [19]. In several studies, it has been reported that fish is one of the main routes of heavy metal exposure to humans [20, 21]. Similarly, human exposure of pesticide residues occurs through fish. Therefore, fish is considered a fresh water system indicator for contaminants and used for assessment of human health risks [22–25]. The contaminants such as pesticide residues can contaminate the aquatic environment, and because of their persistence, bioaccumulation, and toxicity, they may pose serious health threats to humans. The pesticide residues are accumulated in the fish adipose tissues after ingestion from the surrounding environment. The Pangascatfish (*Pangasius pangasius*) is one of the important fish species of Bangladesh. The demand of this kind of fish is increasing day by day in the people of all ages because of its taste and nutritional value. Pangas fish contributes in the annual fish production, and people from rural areas earn their livelihood through fish culturing.

In this current study, we collected pangas fish samples from cultured ponds surrounding Comilla and Mymensingh District. The purpose of the current study is to investigate the residual levels of OPs and CBs in cultured fishes and explore the health risk assessment from fish consumption.

## 2. Materials and Methods

### 2.1. Area of Study

**Figure 1:**
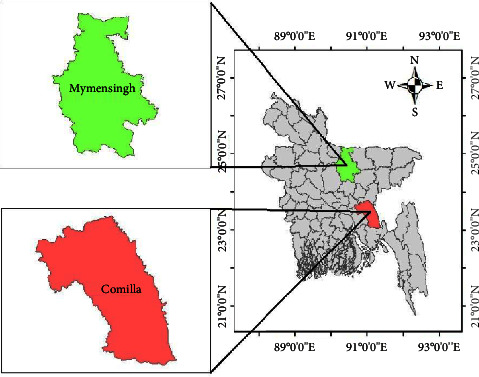
Location map of the study area.

The study was carried out from the cultured Pangas catfish (Pangasius pangasius) from Mymensingh and Comilla area (Figure 1). Mymensingh is located about 120 km north from capital Dhaka. The coordinates of Mymensingh are 24°38′3″N 90°16′4″E. The total area of Mymensingh district is 4363.48 km^2^ with a total population of 5,210,272. The average temperature of Mymensingh is about 12 to 33°C, and average rainfall is 2,174 mm. Comilla is located at the bank of Meghna river. The coordinates of Comilla are 23°27′N 91°12′E. The area of Comilla district is about 3,136 km^2^, while the population of Comilla is 5,387,288. The average temperature of Comilla is 25.5°C, and average rainfall is about 2,295 mm.

### 2.2. Chemicals and Reagents

A cocktail mix containing 8 OP residues (dichlorvos), MOCAP (ethoprophos), disulfoton, methyl parathion, Ronnel, Dursban (Chlorpyrifos), guthion (azinphos methyl), and Tokuthion and a standard mix of 10 CB residues (aldicarb sulfoxide), aldicarb sulfone, oxamyl, methomyl, 3-hydroxycarbofuran, aldicarb, propoxur, carbofuran, Sevin (carbaryl), and methiocarb were purchased from Sigma-Aldrich, USA. Other chemicals including n-hexane, ethyl acetate, acetone, sodium chloride, and anhydrous sodium sulphate were brought from Sigma Aldrich, Germany.

### 2.3. Sample Collection

The American Public Health Association's standard sampling methodology was used to collect 30 surface water samples randomly [26]. This survey was performed from December 2020 to March 2021. Water specimens were taken from the water surface at heights ranging from 30 to 50 cm. To maintain sample quality, samples were preserved in proper conditions after sampling. To study the bioaccumulation of OPs and CBs in fishes, we have used the pangas catfish (*Pangasius pangasius*) as model fish as this fish species cultured widely in Mymensingh and Comilla region. During sampling, fishes of almost same sizes and weights were collected. The collected fishes were stored in the ice box and transported to the laboratory.

### 2.4. Extraction

Pesticide residues were extracted as soon as possible after sample collection (within 24 hours of sample collection). The liquid-liquid extraction (LLE) method has been used to separate OPs and CBs from treated water samples [26]. The OP residues were extracted from fish samples and cleaned up using the QuEChERS extraction method [27]. The collected fish samples were homogenized first, and 10 g of homogenized samples were transferred to a 50 ml centrifuge tube, and 10 mL of acetonitrile was added on it. After vortexing the mix properly, 4 g of magnesium sulphate and 1 g of sodium chloride were added on it and later centrifuged at 5000 rpm for 5 minutes, and the supernatant was collected for the clean-up procedure. In the sample clean-up step, 2 ml of the supernatant was mixed with primary and secondary amine (PSA) (50 mg), graphite carbon black (GCB) (50 mg), and magnesium sulphate (150 mg). When the samples were properly agitated, the samples were centrifuged at 10000 rpm for 5 min and extracts were evaporated through nitrogen stream and reconstituted to 1 ml using toluene prior GC-MS/MS analysis. CB extraction from fish samples was carried out using an ultrasonic extraction process. In brief, 5.0 g of samples of each category were transferred to a 50 mL conical flask. Then, 2.5 g of anhydrous sodium sulphate was added with the samples and mixed to remove the moisture. Later, 20 mL of ethyl acetate was used for extraction through shaking at 270 rpm for 5 minutes. The mixtures were then sonicated at 400°C for 20 minutes using an ultrasonic bath (XUB10, Grant Instruments Ltd., UK) which was followed by standing the mixture for 5 minutes and centrifuging at 2500 rpm for 5 minutes, respectively. The supernatant was collected and concentrated to 1 mL using gentle nitrogen stream. Sample clean-up was performed according to the described process [28]. The extraction cartridges (solid phase) were conditioned by using methanol, distilled water, and ethyl acetate, respectively. Then, a solid phase extraction column was used for sample loading, and samples were eluted with n-hexane: dichloromethane (3:2) mix. Finally, elutes were concentrated using a stream of nitrogen gas, and by using n-hexane, the samples were reconstituted to 1 mL for gas chromatographic analysis.

### 2.5. GC-MS/MS Analysis

The analysis was carried out using a GC chromatograph (Thermo Fisher Scientific, USA) and Trace GOLD TG-5MS GC Column (0.25 mm × 0.25 m × 0.25 *μ*m). The flow rate for this analysis was 1.2 mL/min. The carrier gas was helium. The injection port temperature was 230°C, and the GC temperature ranged from 80°C to 290°C. Sample injection volume was 2 *μ*L. MS analysis was performed through a mass spectrometer (Thermos Scientific, USA).

### 2.6. Method Performance Evaluation

To determine pesticide residues accurately, the suitable approach GC-MS/MS was used. Standard solutions were prepared from pesticide stock solutions. Several dilutions of the standard solution were made ranging from 0.005 mg/L to 0.2 mg/L. LOD and LOQ were determined using the blank solutions. For LOD determination, a signal-to-noise ratio of 3:1 was used, and LOQ detection was performed on 10 times the baseline value of blank samples. The method's accuracy was proven through recovery performance evaluation. Pond water samples were treated with two known quantities for the recovery performance evaluation: 50 *μ*g/L and 100 *μ*g/L.

The samples have been extracted and examined using the following equation:

(1) $$\begin{array}{c} Pi=\left(\frac{Si}{Ti}\right)\times 100\text{,} \end{array}$$

where Pi, Si, and Ti represent recovery percentages, laboratory control results, and percentage recovery of treated samples, respectively.

Matrix effects can suppress or enhance chromatographic signals resulting in low or high residue recoveries. Matrix effects of residues of OP pesticides were estimated in this study according to the following equation:

(2) $$\begin{array}{c} ME\left(\%\right)=\frac{B}{A}\times 100\text{,} \end{array}$$

where *A* and *B* refer to the standard peak area in solvent and standard after spiking, respectively.

The sensitivity of GC-MS/MS analysis was achieved through the use of negative ion chemical ionization. The precursor and product ions were generated upon the tuning of a mass spectrometer. Both precursor and product ions were monitored for each residue of OP and CB pesticides by using the MS1 scan mode. Precursor ions were selected based on the higher abundance and mass-to-charge ratio, while product ions were chosen based on the high abundance of product ions in appropriate collision energy.

### 2.7. Risk Assessment

Risk evaluation with foods and other food-related stuffs where people are exposed indirectly to pesticides was conducted [29, 30]. To know about pesticide residues in daily consumable foods, the estimated daily intake (EDI) will be utilized [31].

The OP and CB strength in the fish and the amount of fish ingested are used to determine the estimated daily intake (EDI) of OPs and CBs. EDIs were computed using the following equation:

(3) $$\begin{array}{c} EDI=\frac{C\times D}{B}\text{,} \end{array}$$

where *C*, *D*, and *B* represent the residual concentrations of OPs and CBs (mg/kg) in fish, daily fish intake (kg/person), and average weight (kg/person), respectively. The daily fish consumption rate of Bangladeshi people is 42.1 g/per capita/day [32]. The average body weight of 60 kg and 16.7 kg for adult people and children was considered for this analysis.

On the contrary, the Hazard Index (HI) is used to estimate the long-term danger of consumers who consume fish with aggregated OPs and CBs in their body. The following formula is used to compute HI from estimated daily intake (EDI) and FAO/WHO recommended average daily intake (ADI) of OPs and CBs:

(4) $$\begin{array}{c} HI=\frac{EDI}{ADI}\text{.} \end{array}$$

When the computed HI value is less than one, consumers are regarded to be safe from any OPs and CB-related health risks [33]. However, if the HI value is greater than one, it can be considered potentially dangerous to one's health.

### 2.8. Data Analysis

Samples were collected in duplicates, and average results were presented as data. The calculations were performed by Microsoft Excel 2010.

## 3. Results and Discussion

### 3.1. Method Performance Evaluation

**Figure 2:**
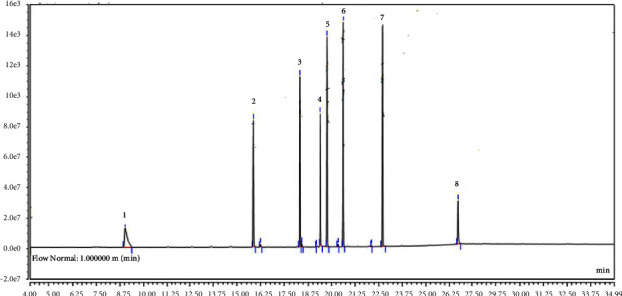
Chromatogram showing peaks of OP pesticide standards. The corresponding peaks of each residue are presented in the following order: 1: dichlorvos; 2: MOCAP (ethoprophos); 3: disulfoton; 4: methyl parathion; 5: ronnel; 6: dursban (chlorpyrifos); 7: tokuthion; 8: guthion (azinphos methyl).

**Figure 3:**
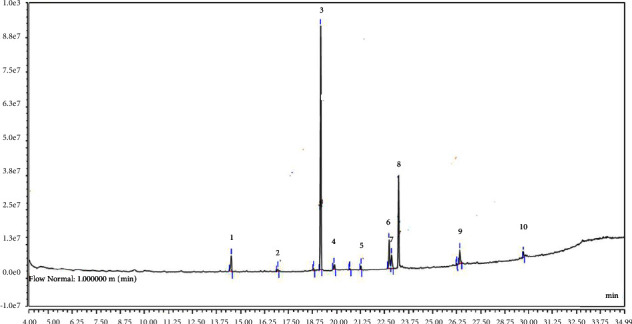
Chromatogram showing peaks of CB pesticide standards. The corresponding peaks of each residue are presented in the following order: 1: aldicarb sulfoxide; 2: aldicarb sulfone; 3: oxamyl; 4: methomyl; 5: 3-hydroxycarbofuran; 6: aldicarb; 7: propoxur; 8: carbofuran; 9: sevin (carbaryl); 10: methiocarb.

The accuracy of the current investigation was evaluated through recovery studies. Tables 1 and 2 represent the elution order, limit of detection, limit of quantification, coefficient of determination (*R*^2^) values, recovery rates, and precursor ion (*m*/*z*) and product ion (*m*/*z*) of OP and CB residues. The percent recoveries were determined between 69.22–94.41% and 66.75–98.65% for OP and CB pesticides, respectively. The coefficient of determination (*R*^2^) varies from 0.9995 to 0.9999 and from 0.9992 to 0.9998 for OP and CB residues, respectively. The LOD values were determined in the range of 0.7–1.0 for CB residues, while LOD values were in the range of 18.32–31.84 *μ*g/L for OP residues. The results of matrix effects of OP pesticide residues are shown in Table 1. The matrix effect of GC-MS/MS analysis followed by QuEChERS extraction occurs in every OP pesticide residues in terms of ionization suppression. Among the pesticide residues, the highest suppression was detected in Tokuthion (27%), while the lowest suppression was detected in disulfoton (8%). The chromatograms showing the peaks of corresponding peaks of OP and CB residues are shown in Figures 2 and 3, respectively.

### 3.2. Presence of OP Residues and CBs in Pond Water Samples of Comilla and Mymensingh

The average OPs and CBs in pond water samples of Comilla and Mymensingh are presented in Tables 3 and 4, respectively. The analysis of carbamate pesticides showed the presence of carbofuran (1.09 ± 0.81 *μ*g/L) and Sevin (carbaryl) (0.78 ± 0.39 *μ*g/L) in pond water samples of Comilla, while only Sevin (Carbaryl) (0.88 ± 0.63 *μ*g/L) was detected from Mymensingh pond water samples. Other carbamate pesticide residues including aldicarb sulfoxide, aldicarb sulfone, oxamyl, methomyl, 3-hydroxycarbofuran, aldicarb, propoxur, and methiocarb were not detected in the water samples of Comilla. Almost similar results were obtained from the water samples of Mymensingh where pesticide residues including aldicarb sulfoxide, aldicarb carbofuran, sulfone, oxamyl, methomyl, 3-hydroxycarbofuran, aldicarb, propoxur, and methiocarb were not detected.

Among the OP compounds, dichlorvos (7.98 ± 3.45 *μ*g/L), methyl parathion (3.87 ± 2.51 *μ*g/L), and Dursban (chlorpyrifos) (5.27 ± 3.56 *μ*g/L) were detected in pond water samples from Comilla, and the presence of OP compounds such as dichlorvos (4.78 ± 3.89 *μ*g/L) and Dursban (chlorpyrifos) (3.82 ± 1.38 *μ*g/L) was detected in the pond water samples of Mymensingh. In that case, the pesticide residues including MOCAP (ethoprophos), disulfoton, Ronnel, Tokuthion, and cushion (azinphos methyl) were not detected in water samples of Comilla, and pesticide residues including MOCAP (ethoprophos), disulfoton, methyl parathion, Ronnel, Tokuthion, and guthion (azinphos methyl) were not detected in the water samples from Mymensingh.

From the current study, the presence of OP and CB residues in pond water samples suggested the use of pesticides in those areas. The presence of these pesticide residues indicated the uses of these pesticides in those areas. The ponds water might be contaminated through the rain water runoff from the agricultural fields. The contamination of fish feed with the pesticide residues might be another reason.

### 3.3. Presence of OP and CB Compounds in Fish Muscle Samples of Comilla and Mymensingh

In order to understand the bioaccumulation of carbamate and OP pesticide residues in fish, the presence of carbamate and OP pesticide residues were detected through GC-MS/MS. The average OPs and CBs in fish muscle samples of Comilla and Mymensingh are presented in Tables 5 and 6, respectively. OP pesticide residues were detected in very low concentrations in fish samples from both of the sampling areas. The fish samples that were collected from Comilla were contaminated with very low concentration of Dursban (chlorpyrifos) (0.88 ± 0.28 *μ*g/kg). On the other hand, fish samples from Mymensingh were contaminated with very low concentrations of dichlorvos (1.17 ± 0.41 *μ*g/kg) and Dursban (chlorpyrifos) (1.04 ± 0.32 *μ*g/kg).

Among the carbamate pesticide residues, only carbofuran (0.68 ± 0.45 *μ*g/kg) in very low concentration was detected in fish samples from Comilla, while only Sevin (Carbaryl) (0.39 ± 0.22 *μ*g/kg) was detected in fish samples from Mymensingh area.

Environmental hazards such as pesticides are one of the prominent threats for all types of people and living organisms. OPs and CBs are the most commonly used pesticides in cultivation. Their improper uses have an influence on the ecosystem. In South Ghana, several pesticides were found to be acute risk to aquatic ecosystems [34]. As shown by European Commission (EC) criteria, the majority of river water samples (93.7 percent) exceeded the EC limit of T-pesticides for drinking of 26 (0.50 ng·mL^−1^) facilitated by pond water samples (56.2 percent) [35]. In Bangladesh, Feni district, the presence of diazinon, carbofuran, and carbaryl pesticide residues in aquatic environment is a severe issue for the ecosystem and heath safety [14]. Another set of findings revealed that the most often identified pesticides in excessive amounts in surface water and sediments were chlorpyrifos, diazinon, and quinalphos [36].

This current study is based on the water and fish samples from cultured ponds in Comilla and Mymensingh that were analysed using the validated method. Tables 3–6 show all of the observed OCPs levels in water and fish samples, respectively. Dichlorvos is different among organophosphates, and in that, it is rapidly metabolized and excreted by mammals [37]. Dichlorvos detoxification mainly occurs mostly in the liver [38]. Dichlorvos causes toxicity by irreversibly inhibiting neural acetylcholine-esterase [39]. Chronic exposure can result in death, as well as genotoxic, neurological, reproductive, carcinogenic, immunological, hepatic, renal, respiratory, metabolic, dermal, and other systemic consequences [40].

In developing countries, methyl parathion (MP) is definitely a more substantial environmental potential hazard. High-risk categories for MP contacts include pregnant women, developing fetuses, infants, growing children, adolescents, and the aged, and the toxicity has been expressed by the tragic incident of MP poisoning in Peru [41]. Methyl parathion poisoning can cause faster, severe organophosphate toxicity and other associated diseases, coma, and death [42].

Epidemiological studies have found statistically significant links between prenatal subacute sensitivity to chlorpyrifos and neurological abnormalities ranging from cognitive deficits to convulsions in children [43]. The young are more vulnerable than adults to the negative effects of chlorpyrifos exposure at levels lower than those which suppress cholinesterase (ChE) [44].

Carbofuran has been implicated to endocrine disruption, reproductive complications, and cytotoxic and genotoxic abnormalities in mammals. Carbofuran is highly detrimental to animals, birds, fish, and wildlife due to its suppression of acetylcholinesterase and butyrylcholinesterase activity [45]. Typically, carbofuran is hazardous to mammals via the oral and breathing pathways but has moderate toxicity via the dermal path. Carbofuran and/or its primary metabolites have the ability to pass the placental barrier and have catastrophic consequences for the maternal-placental-fetal unit [46].

Carbaryl is responsible for the inhibition of FSH-induced progesterone biosynthesis in granulosa-lutein cells [47]. Carbaryl has the greatest possible acute toxicity but the least apoptosis effect of these drugs [48].

Limited studies have been conducted in Bangladesh so far for the identification of pesticide residues in fishes and vegetables [15–17, 49, 50]. In a study in northwestern Bangladesh identified the traces of banned pesticide residues in fish samples [51], while a study on dry barb in the northeastern part of Bangladesh identified OCP residues [52]. The fish samples from Sonargaon upazila contained OCP residues [53].

### 3.4. Human Health Risk Assessment

Human health risks were assessed based on the estimated daily intakes of detected OP and CB pesticides from fish samples. The detected pesticide residual concentrations were compared with the maximum residue level (MRL). Exposure dose was calculated in terms of estimated daily intake (EDI) and the Health Risk Index (HI). The concentrations of both OP and CB residues were detected in the below range than the MRL set by FAO/WHO.

Risk assessment has been carried out for both adults and children. The risk analysis of OPs and CBs through dietary exposure was assessed, and the analysed results are presented in Table 7. The HI values were calculated based on the EDI and ADI values. As we know, an index value larger than 1 indicates risk from consumption of fishes, while an index value smaller than 1 indicates no risks. In this current study, the HI values were determined smaller than 1 after analysis of the fish samples from Comilla and Mymensingh.

From this study, the HI values of Dursban (chlorpyrifos) were detected 6.17467*E*^−05^ and 0.221844311 for adults and children, respectively, in Comilla area. On the other hand, HI values of dichlorvos were detected as 0.000205238 and 0.73738024 for adults and children, while HI values of Dursban (chlorpyrifos) were detected as 7.29733*E*^−05^ and 0.262179641 for adults and children, respectively.

In Comilla, among the detected CB residues, the HI values of carbofuran were 4.77133*E*^−05^ and 0.17142515 for adults and children, respectively. In Mymensingh area, HI values of Sevin (Carbaryl) were 3.42063*E*^−05^ and 0.122896707 for adults and children, respectively.

Considering the current results, the estimated exposure of both OPs and CBs is very low, and this will not pose serious threats to human health after long term exposure.

## 4. Conclusion

The findings of the current study indicated the presence of OPs and carbamate pesticide residues in both pond water and cultured fish samples. The detected values of both OPs and CBs did not exceed the MRLs. Although very low concentrations of pesticide residues were detected in fish samples, their presence in common food such as fish is a matter of great concern. The fish samples from the selected areas are safe for human consumption since the residual levels were below the MRLs fixed by EU/FAO. The health risk assessment study suggested no possible health risks due to fish consumption from the studied area. However, continuous monitoring and control of pesticides uses are strongly recommended in terms of public health safety.

## Figures and Tables

**Table 1:** The elution order, limit of detection, limit of quantification, coefficient of determination (*R*^2^) values, recovery rate, matrix effect (%), precursor ion (*m*/*z*), and product ion (*m*/*z*) of OP residues.

OPP pesticide residues	Elution order	Coefficient of determination (*R*^2^)	Limit of quantification (LOQ) (*μ*g/L)	Limit of detection (LOD) (*μ*g/L)	Recoveries (%)		ME (%)	Precursor ion (*m*/*z*)	Product ion (*m*/*z*)
					50 (*μ*g/L)	100 (*μ*g/L)			
Dichlorvos	1	0.9996	97.15	18.32	77.90 ± 9.17	64.27 ± 6.74	82	183	95
MOCAP (ethoprophos)	2	0.9995	90.28	27.29	69.29 ± 8.67	70.64 ± 9.52	77	244	177
Disulfoton	3	0.9998	55.34	31.84	81.30 ± 6.92	88.26 ± 10.32	92	277	64
Methyl parathion	4	0.9997	70.23	21.23	92.89 ± 6.78	73.19 ± 9.32	90	269	127
Ronnel	5	0.9996	100.62	28.53	88.27 ± 8.39	77.66 ± 7.83	88	234	197
Dursban (chlorpyrifos)	6	0.9997	64.19	31.42	72.43 ± 11.22	69.48 ± 10.11	83	323	127, 1
Tokuthion	7	0.9998	90.22	19.29	69.22 ± 10.43	82.23 ± 9.52	73	308	168
Guthion (azinphos methyl)	8	0.9999	93.43	30.16	94.41 ± 7.63	77.49 ± 8.64	82	321	162, 2

**Table 2:** The elution order, coefficient of determination (*R*^2^) values, limit of detection, recovery rate, precursor ion (*m*/*z*), and product ion (*m*/*z*) of CB analytes.

Analyte	Elution Order	Coefficient of determination (*R*^2^)	Limit of detection	% recovery		Pre cursorin (*m*/*z*)	Production (*m*/*z*)
			(*μ*g/L)	50 *μ*g/L	100 *μ*g/L		
Aldicarb sulfoxide	1	0.9998	1	84.44 ± 5.69	98.65 ± 5.58	209	174
Aldicarb sulfone	2	0.9994	1	88.87 ± 2.90	91.96 ± 4.89	244	144
Oxamyl	3	0.9998	0.8	83.98 ± 3.97	82.56 ± 3.42	167	119
Methomyl	4	0.9997	0.9	91.94 ± 4.97	81.56 ± 4.98	161	111
3‐Hydroxycarbofuran	5	0.9994	1	95.45 ± 5.36	78.54 ± 4.78	239	184
Aldicarb	6	0.9995	0.8	68.53 ± 3.75	67.76 ± 4.97	210	119
Propoxur	7	0.9998	0.9	72.68 ± 4.98	72.27 ± 4.87	227	132
Carbofuran	8	0.9995	0.9	77.54 ± 4.44	70.74 ± 4.68	233	161
Sevin (carbaryl)	9	0.9996	0.7	81.39 ± 4.47	66.75 ± 4.98	208	123
Methiocarb	10	0.9992	0.8	88.98 ± 5.67	70.42 ± 8.74	244	173

**Table 3:** Mean concentration (*μ*g/L) ± SD of detected OP residues in water samples.

OPP pesticide residues	Comilla	Mymensingh
Dichlorvos	7.98 ± 3.45	4.78 ± 3.89
MOCAP (ethoprophos)	ND	ND
Disulfoton	ND	ND
Methyl parathion	3.87 ± 2.51	ND
Ronnel	ND	ND
Dursban (chlorpyrifos)	5.27 ± 3.56	3.82 ± 1.38
Tokuthion	ND	ND
Guthion (azinphos-methyl)	ND	ND

^*∗*^ND: not detected.

**Table 4:** Mean concentration ± SD (*μ*g/L) of detected CBs analytes in water samples.

Analyte	Comilla	Mymensingh
Aldicarb sulfoxide	ND	ND
Aldicarb sulfone	ND	ND
Oxamyl	ND	ND
Methomyl	ND	ND
3-Hydroxycarbofuran	ND	ND
Aldicarb	ND	ND
Propoxur	ND	ND
Carbofuran	1.09 ± 0.81	ND
Sevin (carbaryl)	0.78 ± 0.39	0.88 ± 0.63
Methiocarb	ND	ND

**Table 5:** Mean concentration (*μ*g/kg) ± SD of detected OP residues in *Pangasius pangasius* fish muscle samples.

OPP pesticide residues	Comilla	Mymensingh
Dichlorvos	ND	1.17 ± 0.41
MOCAP (ethoprophos)	ND	ND
Disulfoton	ND	ND
Methyl parathion	ND	ND
Ronnel	ND	ND
Dursban (chlorpyrifos)	0.88 ± 0.28	1.04 ± 0.32
Tokuthion	ND	ND
Guthion (azinphos methyl)	ND	ND

^*∗*^ND: not detected.

**Table 6:** Mean concentration (*μ*g/kg) ± SD of detected carbamate analytes in *Pangasius pangasius* fish muscle samples.

Analyte	Comilla	Mymensingh
Aldicarb sulfoxide	ND	ND
Aldicarb sulfone	ND	ND
Oxamyl	ND	ND
Methomyl	ND	ND
3-Hydroxycarbofuran	ND	ND
Aldicarb	ND	ND
Propoxur	ND	ND
Carbofuran	0.68 ± 0.45	ND
Sevin (carbaryl)	ND	0.39 ± 0.22
Methiocarb	ND	ND

^*∗*^ND: not detected.

**Table 7:** EDI, ADI, and HI values of OPs and CBs in adults and children.

		Mean concentration (mg/kg)	EDI (for adults)	EDI (for children)	ADI (mg/kg/Day)	HI (adults)	HI (children)	Health risk
OPs	Comilla							
	Dursban (chlorpyrifos)	0.00088	6.17467*E*^−07^	0.002218443	0.01	6.17467^*E*−05^	0.221844311	No
	Mymensingh							
	Dichlorvos	0.00117	8.2095*E*^−07^	0.002949521	0.004	0.000205238	0.73738024	No
	Dursban (chlorpyrifos)	0.00104	7.29733*E*^−07^	0.002621796	0.01	7.29733^*E*−05^	0.262179641	No

CBs	Comilla							
	Carbofuran	0.00068	4.77133*E*^−07^	0.001714251	0.01	4.77133^*E*−05^	0.17142515	No
	Mymensingh							
	Sevin (carbaryl)	0.00039	2.7365*E*^−07^	0.000983174	0.008	3.42063^*E*−05^	0.122896707	No

## Data Availability

All data generated or analysed during this study are included within this published article.
